# Global Chromatin Changes Resulting from Single-Gene Inactivation—The Role of SMARCB1 in Malignant Rhabdoid Tumor

**DOI:** 10.3390/cancers13112561

**Published:** 2021-05-23

**Authors:** Colin Kenny, Elaine O’Meara, Mevlüt Ulaş, Karsten Hokamp, Maureen J. O’Sullivan

**Affiliations:** 1 School of Medicine, Trinity College, University of Dublin, Dublin 2, Ireland; ckenny7@tcd.ie; 2 The National Children’s Research Centre, O’Sullivan Research Laboratory, Oncology Division, Gate 5, Children’s Health Ireland at Crumlin, D12N512 Dublin, Ireland; omearaelaine@gmail.com (E.O.); mevlut.ulas@ucd.ie (M.U.); 3 School of Genetics and Microbiology, Trinity College, University of Dublin, Dublin 2, Ireland; karsten.hokamp@tcd.ie; 4 Histology Laboratory, Pathology Department, Children’s Health Ireland at Crumlin, D12N512 Dublin, Ireland

**Keywords:** SMARCB1, rhabdoid tumors, BAF, PBAF, ncBAF, SWI/SNF, tissue differentiation, chromatin remodeling, lineage differentiation

## Abstract

**Simple Summary:**

Malignant rhabdoid tumors (MRT), one of the most lethal, treatment-resistant human cancers, arises in young children within brain, kidney, liver and/or soft tissues. Generally, cancer arises in older adults, and results from *multiple* significant changes (mutations) accumulating in the genetic blueprint (DNA) of a person’s tissues. This blueprint is composed of a 4-letter alphabet. Together, the multiple significant changes in the blueprint then allow a cell to go “out of control”, becoming a cancer cell. The striking thing about MRT is that it has only a single spelling change, so that mutation must be very powerful to lead to such a lethal cancer. Using a model system that we developed, we show herein how this single mutation alters how the whole of the DNA is arranged, thereby having its profound and lethal effects. We present insights into how this mutation arrests maturation of the cells, keeping them in a cancer “state”.

**Abstract:**

Human cancer typically results from the stochastic accumulation of multiple oncogene-activating and tumor-suppressor gene-inactivating mutations. However, this process takes time and especially in the context of certain pediatric cancer, fewer but more ‘impactful’ mutations may in short order produce the full-blown cancer phenotype. This is well exemplified by the highly aggressive malignant rhabdoid tumor (MRT), where the only gene classically showing recurrent inactivation is SMARCB1, a subunit member of the BAF chromatin-remodeling complex. This is true of all three presentations of MRT including MRT of kidney (MRTK), MRT of the central nervous system (atypical teratoid rhabdoid tumor—ATRT) and extracranial, extrarenal rhabdoid tumor (EERT). Our reverse modeling of rhabdoid tumors with isogenic cell lines, either induced or not induced, to express SMARCB1 showed widespread differential chromatin remodeling indicative of altered BAF complex activity with ensuant histone modifications when tested by chromatin immunoprecipitation followed by sequencing (ChIP-seq). The changes due to reintroduction of SMARCB1 were preponderantly at typical enhancers with tandem BAF complex occupancy at these sites and related gene activation, as substantiated also by transcriptomic data. Indeed, for both MRTK and ATRT cells, there is evidence of an overlap between SMARCB1-dependent enhancer activation and tissue-specific lineage-determining genes. These genes are inactive in the tumor state, conceivably arresting the cells in a primitive/undifferentiated state. This epigenetic dysregulation from inactivation of a chromatin-remodeling complex subunit contributes to an improved understanding of the complex pathophysiological basis of MRT, one of the most lethal and aggressive human cancers.

## 1. Introduction

By accepted dogma, cancer is a multistep process requiring the stochastic accumulation of generally 5–6 mutations for the full phenotype with all the pathophysiological “hallmarks” of cancer to be expressed [1,2]. By virtue of this gradual accumulation of mutations over time, human cancer incidence peaks in later adult life. For many decades, all attention in terms of tumor genetic research has been focused almost exclusively on the exome, given that the functional read-out contributing to the cancer phenotype is at the protein level.

An earlier onset of cancer in childhood clearly defies this dogma of stochastic mutation accumulation, with malignant rhabdoid tumors (MRT) representing a remarkable example of this. This tumor of infancy, despite its lethality, bears but one recurrent gene mutation, that of bi-allelic inactivation of the *SMARCB1* (or in rare instances instead, *SMARCA4*) gene [3,4,5]. MRT is otherwise diploid and genomically stable [6].

Admittedly, 35% of patients presenting with MRT have germline mutation of *SMARCB1* (or *SMARCA4*), defining the rhabdoid predisposition syndrome 1 or 2 (RPS1; RPS2), respectively [7,8], but the emergence of a full-blown and indeed highly aggressive cancer so early in life, and hinging on the genomic inactivation of just this one gene, begs the question: what is the normal role of this gene such that its loss of function has such profound and devastating consequences?

*SMARCB1* (also formerly *hSNF5, INI-1, BAF47*) encodes a core protein subunit of the BAF (also SWI/SNF) ATP-dependent chromatin-remodeling complex, which is involved in the energy-dependent (re)organization of chromatin through alterations in the periodicity or torsional stress, and/or histone composition of nucleosomes within the chromatin, contributing in large part to the 5000-fold compaction of DNA within the human nucleus [9,10,11]. In combination with other ‘epigenetic’ mechanisms, such as the covalent modification of particular residues within histone proteins at the core of the nucleosomes, including for example by the Polycomb Repressor Complexes (PRCs), the ATP-dependent chromatin remodelers reversibly (and thus dynamically) regulate not only how DNA is made manifest, but also the recruitment of complexes to the DNA [12,13,14,15], thus having a major role in controlling DNA-templated processes including replication, repair and transcription [16]. In relation to the latter, modification of the chromatin landscape occurs especially at promoters and enhancers [17,18,19]. The BAF complex is necessary for the activation of most promoters and continuously for transcription, however BAF localization is mainly to distal lineage-specific enhancers [10,19].

Herein we sought to establish, by reverse modeling of SMARCB1 loss through the re-introduction of SMARCB1 into malignant rhabdoid tumors cell lines representative of MRTK (G401) and ATRT (BT16), just how dysregulation of the chromatin landscape could contribute to oncogenesis. We focused on a direct comparison of otherwise isogenic cell lines with and without induced expression of SMARCB1, to establish the resultant BAF complex composition and localization, and tandem histone post-translational modifications throughout the genome, with especial reference to regulatory sequences.

As lineage determination and maintenance of cell identity reflect the transcriptome and by inference, any chromatin regulation contributing to this—“cellular state is closely related to chromatin state” [20], we aimed herein to interrogate the cell identity of malignant rhabdoid tumors.

## 2. Results

### 2.1. Restoration of SMARCB1 Leads to Widespread Chromatin Activation in MRT Cell Lines Particularly at Distal Regulatory Elements

**Figure 1:**
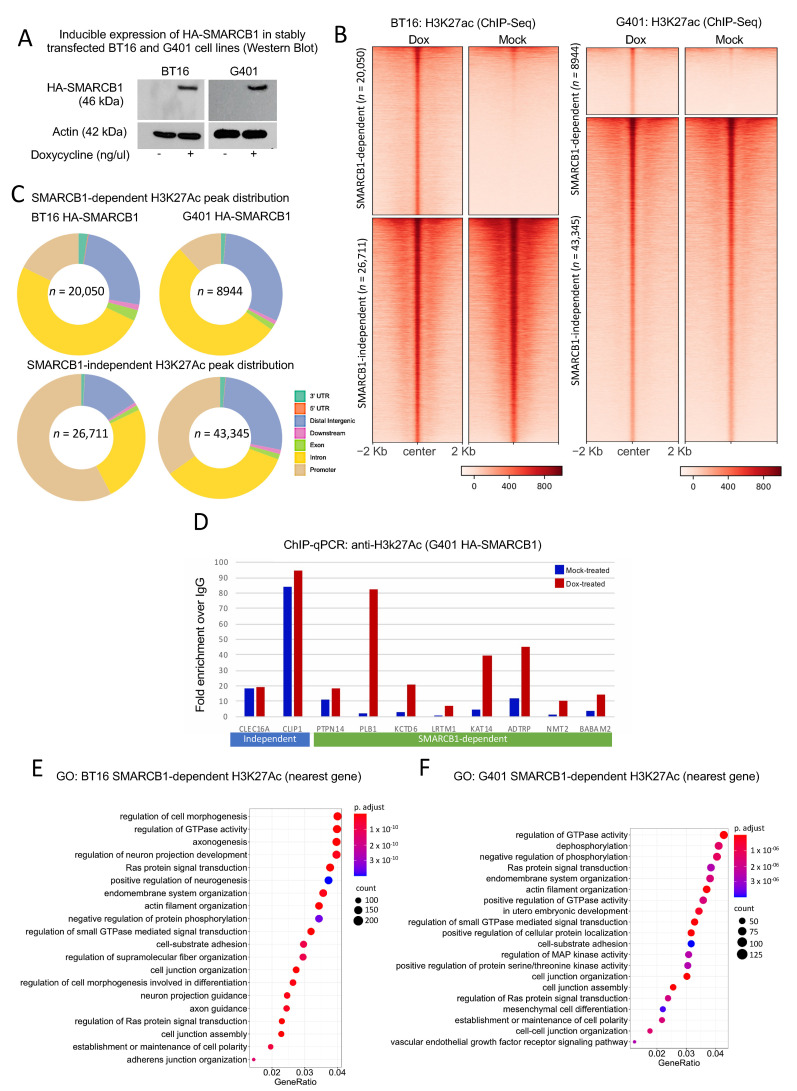
SMARCB1 re-expression leads to global gain of H3K27ac. (**A**) Western blot showing expression of HA-SMARCB1 in dox-treated cells only (Extended blots Figures S1 and S2). (**B**) Density heatmap illustrating 46761 H3K27ac peaks in mock- and doxycycline-treated BT16 cells and 52289 H3K27ac peaks in mock- and doxycycline-treated G401 cells obtained from anti-H3K27ac ChIP-seq. Using MACS2, peaks were grouped into SMARCB1-dependent H3K27ac peaks (*n* = 20,050 for BT16; *n* = 8944 for G401) and SMARCB1-independent H3K27ac peaks (*n* = 26,711 for BT16; *n* = 43,345 for G401). SMARCB1-dependent peaks were identified using a Log Likelihood Ratio (LRR) >2 for BT16 cells and LRR >1 in G401 cells (see methods). SMARCB1-independent H3K27ac peaks showed no H3K27ac signal difference with treatment and were therefore considered common to both treatment conditions. (**C**) Genomic peak distribution of combined SMARCB1-dependent and independent-H3K27ac peaks for each cell line individually are represented by donut plots, indicating most change at distal regulatory elements. (**D**) Chromatin immunoprecipitation qPCR analysis of H3K27ac at SMARCB1-independent peaks (blue underscore) and SMARCB1-dependent peaks (green underscore). Samples were normalized to input and plotted as the fold enrichment over IgG signal. Using ChIPSeeker (association rule, nearest gene within 100 Kb), genes were assigned to SMARCB1-dependent peaks and GO-analysis was performed using ClusterProfiler. (**E**) GO analysis of genes associated with the 20050 SMARCB1-dependent H3K27ac peaks identified in BT16 cells and (**F**) the 8944 SMARCB1-dependent H3K27ac peaks identified in G401. GO analysis is presented by dot plot; adjusted *p*-value is red lowest to blue highest; gene ratio is the ratio between genes associated with a SMARCB1-dependent H3K27ac peak and all genes in the GO category.

SMARCB1 is a core member of the chromatin-remodeling SWI/SNF and PBAF multi-subunit complexes. Various combinatorial permutations exist that are developmental stage and tissue specific and studies have shown that subunit composition can alter gene expression programs during lineage specification and disease [21]. We investigated the contribution of SMARCB1 to changes in chromatin activity in Malignant rhabdoid tumors of kidney (MRTK) and atypical teratoid rhabdoid tumor (ATRT). A panel of cell lines lacking SMARCB1 were considered as model systems for this work. However, the G401 and BT16 cell lines, derived from malignant rhabdoid tumors of kidney and atypical teratoid rhabdoid tumor of the MYC-subtype [22], respectively, and thus inherently lacking SMARCB1 were determined to represent the disease state well, and would provide the most relevant data for investigating MRTK and ATRT-MYC. G401 and BT16 cells were engineered to contain inducible HA-tagged SMARCB1. These cells were treated with doxycycline or mock-treated for 72 h and active histone mark H3K27ac was targeted by ChIP-sequencing. Prior to all ChIP experiments, HA-SMARCB1 expression was confirmed by Western blot analysis (Figure 1A). We performed differential peak analysis using MACS2 and identified sets of peaks that were gained in doxycycline-treated cells (SMARCB1-dependent) and peaks that are common to both treatments (SMARCB1-independent) and as shown by density heatmap of ChIP-signals (Figure 1B). Initial observations suggested that SMARCB1-dependent peaks were distal from the transcriptional start site (TSS) and marked a large set of gained putative enhancers genome-wide (Figure 1C). Noting that SMARCB1-dependent H3K27ac peaks identified in G401 cells were fewer both in number and in read-depth compared to sites identified in SMARCB1-expressing BT16 cell lines, we confirmed the gain of H3K27ac at a selected number of enhancers by ChIP-qPCR (Figure 1D). SMARCB1-dependent H3K27ac sites were associated with biologically relevant GO-terms in their respective tissues (Figure 1E,F). Using proximity rule (nearest gene ±100 Kb), SMARCB1-dependent H3K27ac sites were significantly associated with “axonogenesis” and “positive regulation of neurogenesis”, “neuron projection guidance” and “axon guidance” in BT16 cells and with developmental terms such as “embryonic development” and “mesenchymal cell differentiation” in G401 cells.

### 2.2. SMARCB1-Dependent H3K27ac Peaks Overlap a Subset of Tissue-Specific Enhancers

**Figure 2:**
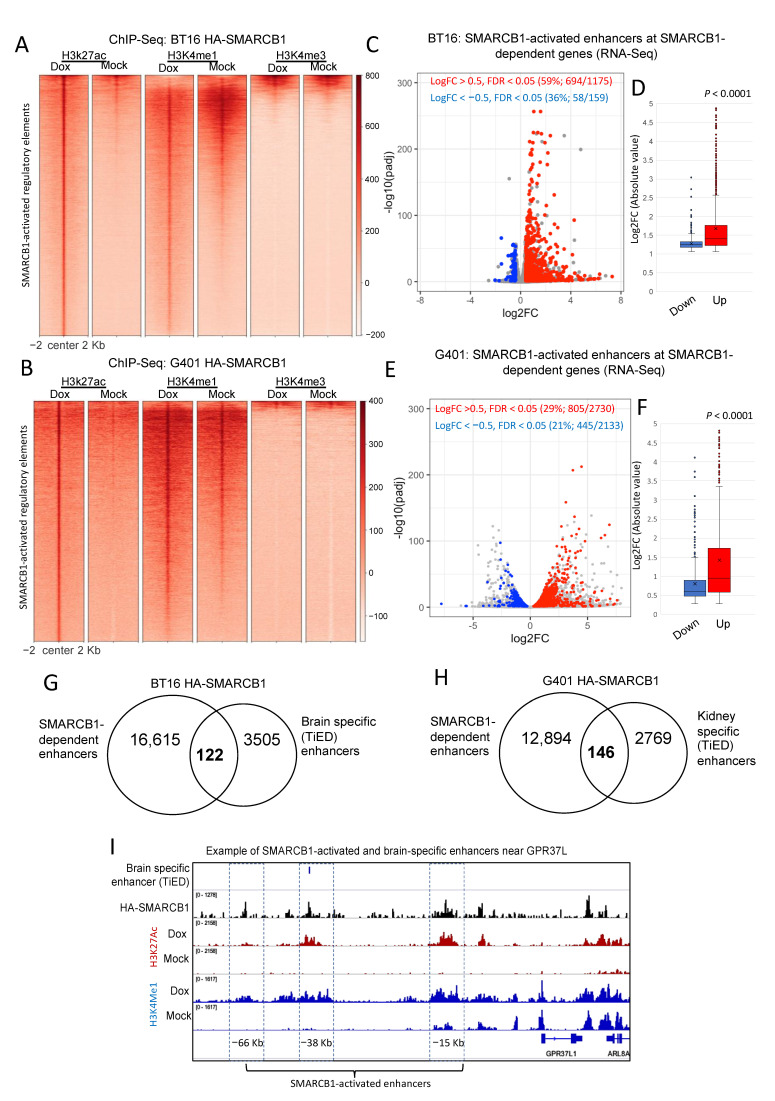
H3K27ac peaks gained in doxycycline-treated cells predominantly mark distal elements and a subset overlap with tissue-specific enhancers. (**A**,**B**) Density heatmaps of SMARCB1-activated enhancers that overlap H3K4me3 and/or H3K4me1, in order to further identify active promoters and enhancers, respectively, in BT16 (**A**) and G401 (**B**) cell lines. Samples are sorted based on doxycycline-treated H3K27ac signal and in descending order of signal strength. Differential analysis of gene expression between mock- and doxycycline-treated BT16 (**C**,**D)** and G401 (**E**,**F**) HA-SMARCB1-expressing cells using RNA-seq online datasets [23,24]. The log10-adjusted value versus the Log2 fold change of expression are plotted. Genes with gained SMARCB1-dependent enhancers and showing statistically significant differential expression (adj *p*-value < 0.05) are highlighted in red (upregulated) or blue (downregulated). The percentage of DEGs that associate with SMARCB1-dependent enhancers in BT16 and G401 cells are represented on the top of each volcano plot. (**D**,**F**) Box-plots representing the absolute log2 fold change of the up- (BT16, *n* = 694; G401, *n* = 805) and down-regulated (BT16, *n* = 59; G401, *n* = 445) genes with a gained SMARCB1-dependent enhancer from (**A**) or (**B**), center line, median; X, mean; box limits, upper and lower quartiles; whiskers, minimum and maximum values; dots, outliers. *p*-value according to the Wilcoxon’s rank sum test is shown. (**G**,**H**) Venn diagrams illustrating the overlap of SMARCB1-activated enhancers from BT16 cells and brain-specific enhancers (*n* = 122) (**G**) and overlap of SMARCB1-activated enhancers from G401 cells and kidney-specific enhancers (*n* = 146) (**H**) as identified from TiED (Tissue Enhancer Database). (**I**) Screenshot of IGV genome browser (GRCH37/hg19), three SMARCB1-dependent enhancers upstream the SMARCB1-activated gene GRP37L1 are shown (painted blue and labelled −66 Kb, −38 Kb and −15 Kb). Dark blue arrows indicate strand orientation and vertical rectangles the exons. Tracks, as labelled include Bed file of brain-specific enhancers (TiED), HA-SMARCB1 ChIP-seq for dox-treated BT16 cells (Black), H3K27ac (Rust) and H3K4me1 (Blue) ChIP-seq for mock- and dox-treated BT16 cells. Y axes are scaled per antibody sample. All three enhancers show gained HA-SMARCB1, H3K27ac and H3K4me1 in dox-treated cells. The +38 Kb enhancer overlaps a brain-specific enhancer (indicated with blue bar, top track).

To functionally characterize H3K27ac gained sites in doxycycline-treated cells, we additionally carried out ChIP-seq using antibodies to chromatin marks characteristic of active promoters (H3K4me3) and active promoters and enhancers (H3K4me1). 2303 (12.7%) and 600 (8%) of the gained H3K27ac peaks overlapped H3K4me3 in BT16 and G401s, respectively, all consistent with activated promoters, whereas a larger fraction of gained H3K27ac peaks, 17,747 (87.3%) and 8344 (92%) in BT16 and G401s, respectively, overlapped H3K4me1 without H3K4me3, consistent with activated enhancers. While we did observe greater H3K27ac signals at promoters upon reintroduction of SMARCB1, we did not observe a concomitant change in the level of H3K4me3, which was strong in both mock- and doxycycline-treated cells, suggesting that H3K4me3 deposition is independent of SMARCB1 at this subset of promoter peaks. Enhancer regions on the other hand were strongly positive for H3K4me1 in tandem with gained H3K27ac in the doxycycline-treated cells (Figure 2A,B). These results suggest that SMARCB1 is a key regulator of enhancer activation. Next, we examined published RNA-seq profiles from BT16 and G401 cells expressing SMARCB1 [23,24]. We hypothesized that many of the genes altered by SMARCB1 expression would harbor SMARCB1-activated enhancers (proximity ±100 kb of a TSS) and that these genes would be associated with growth and differentiation. In BT16 cells, 59% (694/1175) of the upregulated genes and 36% (58/159) of downregulated genes (FDR < 0.05 and |log2FC| > 0.5) were associated with one or more gained active enhancer (hypergeometric test 1.2 × 10^−33^). We similarly profiled dysregulated genes in doxycycline treated G401 cells, to find that 29% (805/2730) of the upregulated genes and 21% (445/2133) of the downregulated genes were significantly associated with SMARCB1-activated enhancers (hypergeometric test 9.8 × 10^−76^). SMARCB1-dependent enhancers significantly associated not only with SMARCB1-activated genes (i.e., upregulated genes in dox-treated cells) but also with SMARCB1-suppressed genes (i.e., downregulated genes in dox-treated cells), implying that SMARCB1 can remodel chromatin and facilitate the binding of both transcriptional activators and repressors. Nevertheless, the average fold change of the upregulated transcripts was significantly higher than that of the downregulated ones (Figure 2C–F), suggesting a higher global impact on positively regulated genes upon SMARCB1 rescue in BT16 and G401 cell lines. Using PANTHER classification system, the SMARCB1-dependent upregulated genes were significantly associated with developmental gene ontology terms including, but not limited to, “neurogenesis (GO:0022008)”, “neuron differentiation (GO:0030182)” and “generation of neurons (GO:0048699)” in BT16 cells and with “kidney development GO:0001822” and “cell proliferation involved in kidney development GO:0072111” in G401 cells.

Given that mock-treated G401 and BT16 cells, respectively, model the cancerous state of MRTK and ATRT, we reasoned that the cells of these primitive undifferentiated tumors might lack tissue-specific regulatory cues necessary for lineage specification and terminal differentiation. To test this, we first examined clusters of narrowly spaced enhancers, called stretch- or superenhancers (SEs), in mock- and doxycycline-treated cells. SEs are linked to cell type-specific gene expression and newly acquired super-enhancers at oncogenes can lead to tumorigenesis [25]. Following published methods, we used H3K27ac ChIP-seq data and identified a total of 967 and 970 SEs in mock- and doxycycline-treated G401 and BT16 cells, respectively. As expected, the level (read-depth) of H3K27ac was remarkably strong at SEs. However, we observed no significant change in signal strength upon SMARCB1 induction (Figure S3 in G401 or BT16 cells, suggesting that these events are less likely to be contributors to MRT development.

Interestingly, we identified 122 regions that are both gained in SMARCB1 re-expressing BT16 cells and classified as brain-specific regulatory elements (corresponding to typical enhancers) by TiED [26] and 146 TiED kidney-specific enhancers in SMARCB1 re-expressing G401 cells (Figure 2G–H). When comparing our SMARCB1-activated enhancers with tissue-specific enhancers (see methods), brain and kidney remained amongst the top-most enriched tissue types when compared with SMARCB1-activated enhancers from BT16 and G401 cells, respectively. An example of a SMARCB1-dependent, brain-specific enhancer near the brain related gene GPR37L is shown (Figure 2I). GPR37L is upregulated in dox-treated BT16 cells, here we demonstrate that the level of H3K27ac and H3K4me1is reduced at three enhancers (−66 Kb, –38 Kb and −15 Kb) upstream of GPR37L in mock-treated cells and gained in the dox-treated cells. The associated occupancy of these elements by HA-SMARCB1 upon doxycycline treatment demonstrates that these are indeed directly SMARCB1-activated regulatory regions. The –38 Kb putative enhancer is identified as a brain-specific enhancer by TiED. These findings support our hypothesis that MRT develops due to the loss of activation of tissue-specific enhancers as a result of SMARCB1 loss, and thereby failure to activate cell/tissue differentiation programs.

### 2.3. Re-Expression of SMARCB1 Leads to Increased SWI/SNF Complex Occupancy at Distal Enhancers

**Figure 3:**
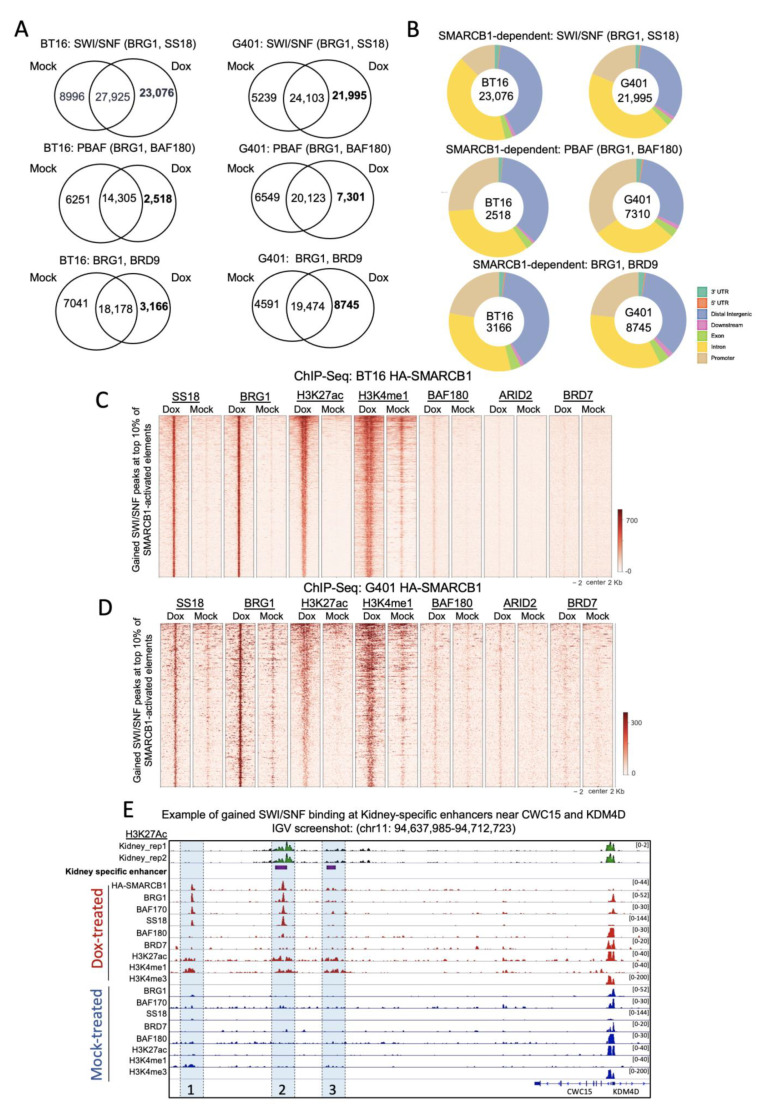
Loss of SMARCB1 alters SWI/SNF binding at typical enhancers. (**A**) Venn diagram illustrating SWI/SNF, PBAF, and ncBAF binding events in mock- and doxycycline-treated cells. (**B**) Donut plots illustrating peak distribution of gained SWI/SNF, PBAF and ncBAF complexes in doxycycline treated BT16 and G401cell lines. (**C**,**D**) Density heatmaps of BAF complex binding, grouped according to BRG1 (core), SS18 (BAF), H3K27ac, H3K4me1, BAF180 (PBAF), ARID2 (PBAF) and BRD7 (PBAF) at the top-10% of activated H3K27ac elements in mock- and doxycycline-treated cells. Samples are sorted based on doxycycline-treated H3K27ac signal and in descending order of signal strength. (**E**) Screenshot of IGV genome browser at a region on chromosome 11. Three gained enhancers are shown (painted blue and labeled 1–3) with nearby genes *CWC15* and *KDM4D*. Dark blue arrows indicate strand orientation and vertical rectangles the exons. Tracks, as labelled include H3K27ac ChIP-seq for normal kidney (in green) and HA-SMARCB1, BRG1, BAF170, SS18, BAF180, BRD7, H3K27Ac, H3K4Me1 and H3K4Me3 ChIP-seq for doxycycline- (in red) and mock-treated (in blue) G401 HA-SMARCB1 cell lines. Y axes are scaled per antibody sample. The first enhancer shows gained occupancy of SWI/SNF complex members, HA-SMARCB1, BRG1, BAF170 and SS18 in doxycycline-treated cells. The second gained enhancer overlaps a kidney-specific enhancer (indicated with purple bar) and shows gained occupancy of SWI/SNF in SMARCB1 re-expressing cells. The third gained enhancer also overlaps a kidney-specific enhancer. However, no binding of SWI/SNF was observed at this region. The level of H3K27ac and H3K4me3 ChIP-seq signal was unchanged at the promoters of both genes.

To study the effect of SMARCB1 re-expression on BAF complex composition and genome-wide occupancy, we targeted core subunits (BAF170, BAF155, BRG1, HA-SMARCB1), SWI/SNF (SS18, SS18L), PBAF (ARID2, BAF180, BRD7) and ncBAF (BRD9) [27,28] subunits by ChIP-seq. Given our findings that BT16 and G401 cells show apparent loss of active brain- and kidney-specific enhancers, respectively, we reasoned that rescue of SMARCB1 would re-direct the BAF complex(es) in both BT16 and G401 cells to enhancers of differentiation genes important in brain and kidney development, respectively, that are aberrantly lost during tumorigenesis. To test this, we compared binding events in mock- and doxycycline-treated cells. SWI/SNF targets were defined as the overlap of core subunits (BRG1, BAF155, BAF170) with SS18. PBAF targets were defined by the overlap of core subunits with BAF180, ARID2 and BRD7; and ncBAF targets, by the overlap of core subunits (BRG1 and BAF155) with BRD9. In both BT16 and G401 cell lines, we observed the highest number of gained peaks for SWI/SNF upon SMARCB1 induction with far lesser gained peaks for PBAF or ncBAF (Figure 3A). The doxycycline gained peaks localized distal to the TSS compared to common and mock-specific peaks and this trend was strongest for the SWI/SNF complex in both cell lines (Figure 3B and data not shown) [29]. 

Dox-gained SWI/SNF targets, both proportionally and absolutely, far outnumbered gained-PBAF or -ncBAF targets, suggesting overall that SMARCB1 loss of function has a particularly profound effect on SWI/SNF occupancy, and particularly so at enhancers (Figure 3A,B).

Next, we overlapped these gained SWI/SNF, PBAF or ncBAF peaks with SMARCB1-dependent H3K27Ac peaks to identify regulatory regions that are active and occupied by chromatin remodelers in SMARCB1 re-expressing cells. We focused on the top 10% of SMARCB1-dependent H3K27ac peaks in doxycycline-treated cells (i.e., greater read-depth over mock-treated cells). In BT16 cells, the gained SWI/SNF peaks overlapped 54% of SMARCB1-activated elements. In G401s, a lower fraction (21%) of SMARCB1-activated elements were occupied by gained SWI/SNF peaks, however similarly to BT16, it was SWI/SNF predominantly rather than PBAF that showed a dox-gained occupancy at these sites (Figure 3C,D). Examples of activated kidney-specific enhancers with a gain of SWI/SNF binding near CWC15 and KDM4D genes showing multiple subunits of SWI/SNF occupying these enhancers in dox- but not mock-treated cells (Figure 3E).

These aggregate results suggest that SMARCB1, as a fundamental subunit of the SWI/SNF complex, is a major contributor to the targeted activation of distal enhancers in both BT16 and G401 cell lines.

### 2.4. BRD9 and BRG1 Contribute to Cell Survival in BT16 and G401 Cell Lines

**Figure 4:**
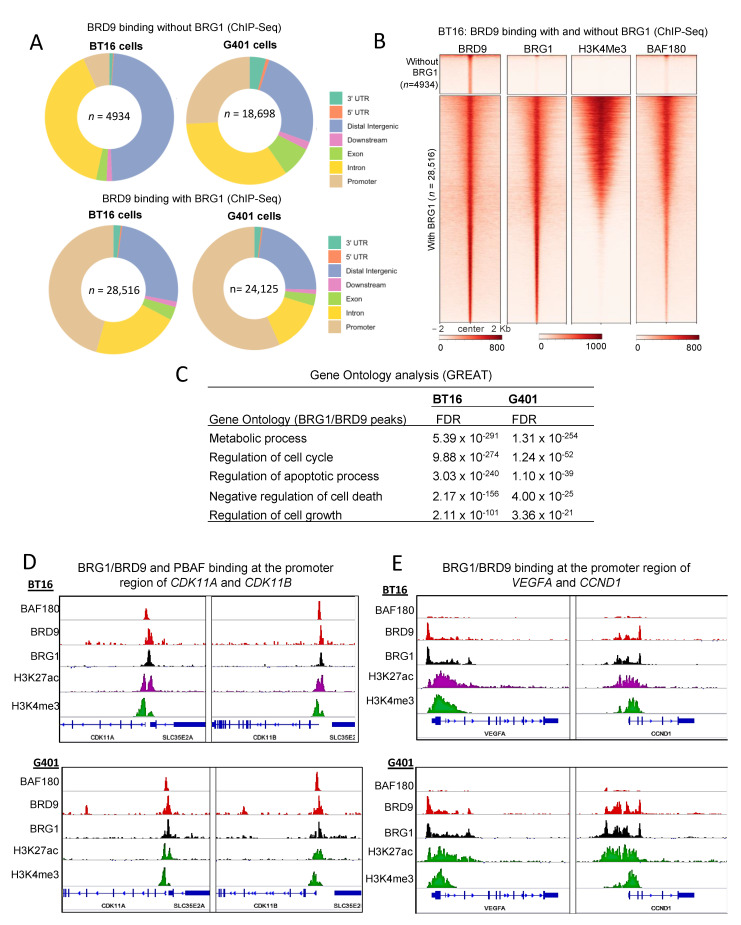
Characterization of BRD9 and BRG1 binding in mock-treated BT16 and G401 cell lines. (**A**) Donut plots BRD9 and BRG1 peaks in mock-treated G401 and BT16 cell lines were intersected using BEDtools and donut plots representing peak distribution were generated. (**B**) Density heatmap illustrating two groups (Top) 4934 BRD9 peaks without BRG1 binding and (Bottom) 28,516 BRD9 peaks with BRG1 binding in mock-treated BT16 cell lines. The heatmap is grouped according to BRD9, BRG1, H3K4me3 and BAF180. Samples are sorted based on BRD9 signal and in descending order of signal strength. (**C**) GREAT analysis of BRG1/BRD9 peaks in mock-treated BT16 and G401 cells. (**D**) Screenshot of IGV genome browser (GRCH37/hg19) showing positive ChIP-seq signal for BAF180, BRD9, BRG1, H3K27ac and H3K4me3 at the promoter region of *CDK11A* and *CDK11B* in BT16 and G401 cell lines. (**E**) Screenshot of IGV genome browser (GRCH37/hg19) showing positive ChIP-seq signal for BRD9, BRG1, H3K27ac and H3K4me3 at the promoter region of *VEGFA* and *CCND1* in BT16 and G401 cell lines. Dark blue arrows indicate strand orientation and vertical rectangles the exons. Y axes are scaled per antibody sample. Normalization RPKM.

Given that rhabdoid tumor cell lines are susceptible to BRD9 inhibition [30,31], we sought to investigate the direct targets of BRD9 in mock-treated cell lines (which model the cancerous state) by ChIP-seq. We first overlapped BRD9 peaks with core subunit BRG1 to identify 28,516 and 24,125 overlapping sites in BT16 and G401 cells, respectively (Figure 4A). A subset of BRD9 peaks in both cell lines did not overlap BRG1 (Figure 4A,B top panels of heatmap). Proportionally more of these were located distally from a TSS (Figure 4A) with a shift to more promoter occupancy for the co-localized BRD9/BRG1, and with these promoter regions marked also with H3K4me3 (Figure 4A,B lower panels of heatmap).

Given the exquisite sensitivity of SMARCB1-deficient cells to BRD9 inhibition [30,31], we hypothesized that BRD9/BRG1 peaks would occupy activate promoters associated with the hallmarks of cancer. In both cell lines, these sites were predominantly identified at promoters marked with H3K4me3. Notably a considerable fraction of BRD9/BRG1 peaks were also identified at distal elements. Moreover, we observed co-occupancy of BAF180 (PBAF) at BRD9/BRG1 targets also, suggesting that PBAF and BRD9/BRG1 complex members might co-localize to such sites in mock-treated cells. Co-localization of BAF180 with BRD9/BRG1 at promoters and enhancers was previously reported [28,30,31], which is consistent with our observations (Figure 4B). Motif analysis for transcription factor binding sites at BRG1/BRD9 targets showed strong enrichment for the binding motif of CTCF, FLI1, ETV4 and YY1 ( Tables S1 and S2). GREAT analysis of the BRD9/BRG1 co-occupied peaks shows involvement in metabolism, cell cycle and pro-survival, including negative regulation of cell death (Figure 4C). Examples of BRG1/BRD9 binding at active promoters (H3K4me3 and H3K27ac) of *CDK11A*, *CDK11B, CCND1* and *VEGFA* are shown in Figure 4D,E for both G401 and BT16 cell lines. BRG1/BRD9 and PBAF co-occupy the promoters of pro-survival genes *CDK11A* and *CDK11B*, whereas only BRG1/BRD9 peaks were identified at the promoters of *CCND1* and *VEGFA* in BT16 and G401 cell lines.

### 2.5. DNA Methylation May Contribute Additional/Alternative Means of Repressing Developmental Gene Expression in MRTK Tumors

**Figure 5:**
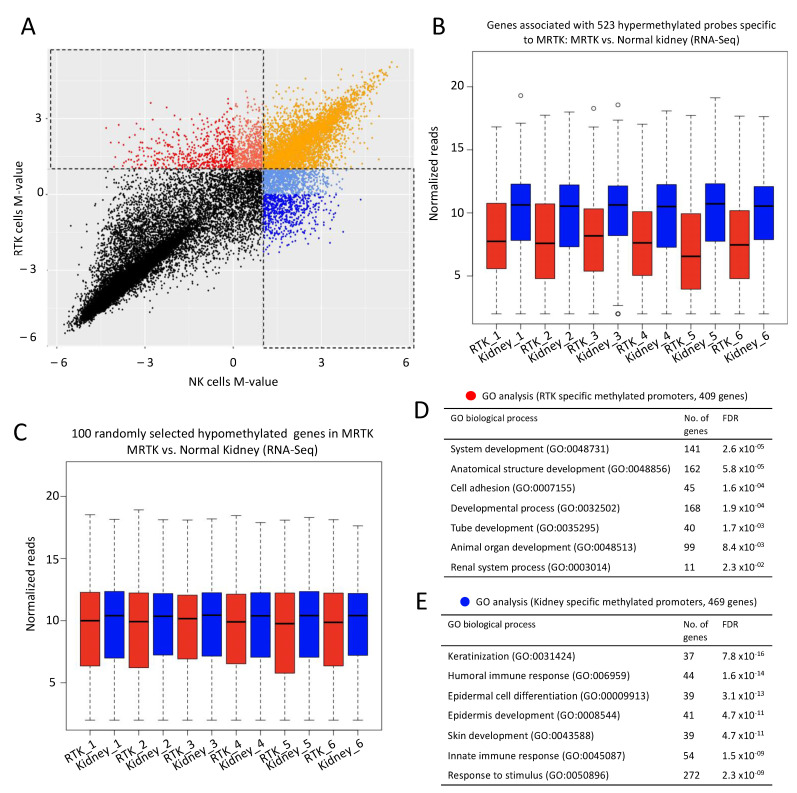
DNA methylation at promoters of developmental genes in rhabdoid tumors may provide additional/alternative means of repression of lineage differentiation (**A**) Scatterplot represents DNA methylation data from non-neoplastic kidney (NK; *n* = 3) and rhabdoid tumors of the kidney (MRTK; *n* = 3). Methylation signal intensities were converted to M-value by transferring the ratio of methylated vs. unmethylated signal to log2 scale. Probes with high M-value in MRTK samples and low M-value in NK samples are shown in Red (*n* = 523, >2 fold methylation in MRTK) and probes with low M-value in MRTK samples and high M-value in NK samples are shown in Blue (*n* = 642, >2 fold methylation in NK). Additional probes include tomato red (*n* = 569, >2 fold methylation in MRTK and <1 fold methylation in NK), cornflower blue (*n* = 961, >2 fold methylation in NK and <1 fold methylation in MRTK) and orange (*n* = 4036, >2 fold methylation in both MRTK and NK). Each probe was assigned to its nearest gene ±5 Kb and publicly available RNA-seq datasets from six MRTK and matched normal kidney samples were used to determine the expression levels of genes with hypermethylated promoters specific to MRTK tumors compared to normal kidney. Genes with MRTK hypermethylated promoters (associated with 523 probes) (**B**) and 100 randomly selected genes (**C**), red = MRTK samples, blue = NK samples, *y*-axis log10 normalized reads. (**D**,**E**) Genes whose promoter CpG islands showed methylation specific to MRTK were significantly associated with developmental programs and cell adhesion whereas promoter CpGs showing methylation in normal kidney were associated with silencing of epidermal cell differentiation, skin development and innate immune response. A full list of probes with M-values for MRTK and NK can be found in Tables S3 and S4. Publicly available Methylation and RNA-seq datasets from [33,34].

DNA methylation at CpG islands is an important regulator of gene expression. Due to CG suppression (where cytosine is spontaneously converted to thymine), CpG islands are selectively found at promoters. In fact, in humans approximately 70% of promoters contain a CpG island [32]. Methylation of CpG islands is a well-accepted mechanism of gene silencing and alterations to methylation programs have been implicated in cancer. In line with our findings on the global chromatin inactivation in MRTK, we hypothesized that CpG island methylation may provide an alternative/additional means of lineage-specific gene suppression in MRTK. We analyzed publicly available DNA methylation data from three normal kidney (NK) and three MRTK samples [33]. The majority of probes were within 2 kb of the TSS, thus simplifying the gene association rule for downstream analysis. To detect regions that are strongly methylated in tumors or kidney samples, we plotted the methylation signal vs. unmethylation signal for each probe from MRTK against that from NK. We then selected probes with log2 ratios greater than 1 in MRTK and less than 0 in NK (Figure 5A). We identified 523 probes that are strongly methylated in MRTK and 642 probes that are strongly methylated in NK. To then correlate CpG promoter hypermethylation with gene expression we utilized publicly available RNA-seq datasets from six MRTK tumors and matched normal kidney tissue [34]. We hypothesized that hypermethylated genes that where specific to MRTK (i.e., the genes that associated with the 523 methylated probes specific to MRTK) would show significantly reduced mRNA message levels compared to normal kidney. Indeed, our analysis confirms the relatively lower expression (repression) of genes with CpG island promoter hypermethylation in MRTK compared with normal kidney (Figure 5B). This was not a random observation as the expression levels of 100 randomly selected genes without hypermethylation in MRTK showed no significant expression difference when compared with normal kidney (Figure 5C). Using GO-analysis, we found that the methylated promoters in MRTK were strongly associated with cell differentiation, including kidney developmental processes and differentiation genes. We speculated that amongst genes with methylated promoters in normal kidney would be those involved in alternative lineage determination, thus repressing genes not related to kidney development. Methylated promoters identified in normal kidney were strongly associated with keratinization, immune-response, epidermal differentiation and skin development but completely lacked any association with renal differentiation processes (Figure 5B).

## 3. Discussion

While cancer genetic research has focused very extensively on the exome over recent decades, given that mutations within coding sequence may clearly impact significantly on the protein products of these genes to contribute to oncogenesis, it has emerged more recently that dysregulation at an epigenetic level, resulting for example from mutation in subunits of the BAF complex, may in fact contribute considerably to cancer development as well as progression [35,36,37,38,39,40]. Changes to the chromatin are possible by altered spacing of nucleosomes through the actions of ATP-dependent chromatin-remodeling complexes such as BAF, and/or covalent modification of histone protein tails by enzymes such as EZH2, the catalytic subunit of the PRC2 complex. Nucleosome depletion tends to parallel gene activation [41] and so, dynamic but highly regulated alterations of chromatin account for the diversity of transcriptome, defining our >200 diversely specialized human cell types, despite all these cells (in the absence of mosaicism) sharing a common germline DNA. Indeed, the orchestrated modification of chromatin of totipotent cells permits the activation of pluripotent lineage-determining genes, contributing also to their ongoing expression in terminally differentiated cells for cell identity maintenance throughout life, while simultaneously silencing genes defining alternative lineages [20,42,43,44,45]. Cis-regulatory elements including promoters, enhancers and insulators are important in this, but especially enhancers are key to cell fate determination [46]. Chromatin signatures are far more variable at enhancers across different cell types than at promoters. So-called ‘poised’ enhancers—often already looped to promoters of key early developmental genes and bearing combinations of repressive and active marks early in embryogenesis—undergo ‘resolution’ by losing repressive marks and gaining activation marks with lineage specification [20,46]. Thus, chromatin modifications regulate enhancers by contributing to assembly of transcriptional complexes, promoting accessibility for transcription factors, priming regulatory elements for further use later, and through long-range communication with promoters.

The importance of the BAF complex in development/differentiation has long been recognized in Drosophila, where *Hox* gene repression is maintained by Polycomb group proteins (PcGs) while the BAF complex promotes *Hox* gene activation, in a dynamic and mutually antagonistic fashion [47,48,49,50]. BAF is highly conserved throughout eukaryotes [46,50] having first been identified in yeast [47,51], where the complex fails to form in the absence of SMARCB1 [52]. This is not the case in humans, where chromatin remodeling indeed hinges on the ATPase alone [53] and where complex formation can proceed in the absence of SMARCB1 [40,53] but with experimental evidence of some reduced affinity chromatin binding in this context [23,24]. Several cell- and context-dependent BAF complexes exist, the best established constellations in mature cells being BAF and PBAF, which share three essential core subunits [53] along with 7–15 variable subunits [27,54,55,56,57,58]. A more recently recognized non-canonical ncBAF complex contains BRD9, which is more avidly incorporated into complex formation in the absence of SMARCB1 and which is indeed required for cell survival in that context [31]. BAF180, BAF200, BAF45A and BRD7 are found in PBAF but not BAF, whereas SS18, BAF250A/B and BAF45D are components of BAF but not PBAF [59]. Interestingly, these subunits show mutation recurrently in certain human cancer subtypes, reiterating their cell- and context-specificity [59].

Most BAF subunits can bind DNA; bromodomains within ATPases bind acetylated histone lysines, chromodomains of BAF155 and BAF170 have affinity for methylated histones, not to mention the recruitment of BAF by transcription factor binding [60]. Importantly, more than 90% of BAF subunit binding is at functional regions including the 5′ end of protein coding genes, RNAPol II regions, CTCF sites or enhancers. These regulatory regions are now well defined through a combination of localization relative to identifiable features such as transcription start sites, or in the case of enhancers by their transcription factor binding motifs in addition to histone tail covalent modifications marking their ‘active’ state [61]. By these features, so-called ‘superenhancers’ are also separated out as those having exceptionally high transcription factor binding density coupled with larger and broader histone modification profiles in chromatin immunoprecipitation studies, and an order of magnitude greater size than ‘typical’ enhancers [25]. Superenhancers are implicated in the control of master regulators of cell identity. The BAF complex binds to ~33% gene promoters and also to active enhancers and in the context of SMARCB1 inactivation, there results dysregulation of 1300 genes including multiple cancer-associated pathways such as CCND1/CDK4, SHH, and WNT/B-Catenin—in essence too many to contemplate therapeutic targeting [62,63,64].

Early investigations of malignant rhabdoid tumors showed that re-expression of SMARCB1 led to activation of transcription initiation either by recruitment of BAF complexes or activation of existing ones, implying that oncogenesis in the context of SMARCB1 loss is due to promoter pausing [64]. Genome-wide ChIP-sequencing studies, however, revealed that chromatin modification in this setting is really widespread, with our data showing 20,050 and 8944 high-confidence H3K27ac peaks gained upon SMARCB1 re-expression in BT16 and G401 cell, respectively, and these occurring especially at enhancer sites, a finding also shared by others [24,65]. These profound gains of H3K27ac at enhancers are accompanied by a somewhat less striking gain of H3K4me1 activation marks at these enhancers and the observation applies to typical enhancers rather than superenhancers (our data and [24]).

Although enhancers are key to cell fate determination, superenhancers are considered crucial notably in the regulation of master oncogenes, but no significant findings emerged from our data in terms of superenhancers specific to ‘mock-treated’ MRT cells that are then ‘lost’ with re-expression of SMARCB1 and which might thereby elucidate oncogenes specifically important to MRT establishment and survival. Some insights into the ability of the cancer cell (modeled here with the mock-treated cell lines) to survive and proliferate—starkly contrasted by the cellular senescence within few days following SMARCB1 rescue—came instead from our investigation into the role of ncBAF subunit BRD9 in the mock-treated cells. In the absence of SMARCB1, relatively larger proportions of BRG1 are incorporated into PBAF and ncBAF complexes than in the presence of SMARCB1. ncBAF complex, characterized by BRD9 amongst other subunits, has been reported to localize to CTCF and promoter loci [65]. This was very clearly the case from our data too. In the mock-treated (cancerous) model, BRD9 together with BRG1 localizes preferentially to promoters that are marked as active by H3K4me3 including those involved in pathways important for cell survival, which is a provocative finding in light of the recently reported exquisite vulnerability of SMARCB1-deficient cells to BRD9 inhibition [30]. We find that PBAF co-localizes with BRD9/BRG1 at some but not all active promoters in this context also, as have others [31].

We show herein overlaps between activation and tissue specificity of enhancers for both cell lines. Indeed, amongst top tissue specificity of doxycycline-gained enhancers for G401 and BT16 cell lines are kidney and brain, respectively. Furthermore, analysis of DNA methylation in tissue samples from normal kidney and from malignant rhabdoid tumors of kidney reveals evidence of suppression of renal differentiation-associated genes in MRTK and by contrast, silencing of ‘alternative’ differentiation lineages in normal kidney tissue at promoter CpG islands, the bona fide epigenetic mechanism of gene silencing. Taken together, these observations support our contention that failure of differentiation underpins the oncogenic process in MRT development.

## 4. Materials and Methods

### 4.1. Cell Line Generation

The G401 and BT16 cell lines were stably transfected with doxycycline-inducible HA-tagged *SMARCB1* following a previously published protocol [66] with minor modifications. *SMARCB1* was amplified from normal kidney tissue and cloned with HA-tag into a doxycycline/tetracycline-inducible pLVX Tight Puro lentiviral (Clontech, Mountain View, CA, USA) expression system. Cells were transfected with viral packaging vector Pax2, envelope vector MD2G, and pLVX Tight Puro containing HA-SMARCB1 using Lipofectamine 2000 (Thermo Fisher Scientific, Waltham, MA). The optimum dose of doxycycline to induce HA-SMARCB1 expression was determined as 0.1 ng/μL. SMARCB1 protein expression was verified by Western blot analysis prior to each experiment.

### 4.2. Tissue Homogenization

Normal kidney tissue samples were identified by a consultant pathologist, retrieved fresh and snap-frozen and stored at −80 °C without delay following nephrectomy for tumor resection. Samples were anonymized and had been obtained with patient/parental consent providing for use in biological studies. A relevant application passed through the Medical Research Ethics Committee of Children’s Health Ireland at Crumlin where approval was granted. Snap frozen kidney tissue (0.1 g) was cut into small pieces using a sterile surgical scalpel. Cut tissue was transferred to 15 mL tubes containing ice-cold PBS. The cut tissue was homogenized using a TissueRuptor II (Qiagen, Germantown, MD, USA). Cells were centrifuged for 5 min at 600× *g*, cell pellets were washed twice in PBS and crosslinked as described below.

### 4.3. One- and Two-Step Crosslinking Reaction

Standard one-step crosslinking was performed when targeting histone post-translational modifications (H3K4me3, H3K27ac, H3K4me1 and H3K27me3) for ChIP-sequencing. G401 and BT16 HA-SMARCB1 cells were cultured in T75 flasks (Thermo Fisher Scientific, Waltham, MA, USA) and treated with 0.01 ng/uL doxycycline or DMSO (mock treatment) for 72 h. The cell monolayer or homogenized kidney tissue was washed twice with pre-warmed PBS. Cells were incubated at room temperature in 1% formaldehyde (FA) solution (Millipore Sigma, Burlington, MA, USA) for 10 min before adding 0.125 M glycine (Millipore Sigma) to quench the crosslinking reaction. Two-step crosslinking [67] was performed to target SWI/SNF subunits; BAF170, BAF155, BRG, SS18, ARID1A, BRD9, BAF180, ARID2, BRG7 and HA-SMARCB1 for ChIP-sequencing. The 2 mM disuccinimidyl glutarate (DSG; Thermo Fisher Scientific, Waltham, MA) was added to cells for 30 min at room temperature. Excess DSG was removed by washing twice in PBS and cells were crosslinked with FA solution for 10 min as described above. Crosslinked cells were washed twice in PBS and lysed in 5 mL SDS lysis buffer (100 mM NaCl, 50 mM Tris-Cl, 5 mM EDTA, 0.02% NaN^3^, and 1% SDS) containing protease inhibitors (1 μg/mL leupeptin, 1 μg/mL aprotenin, 10 μM PMSF; Millipore Sigma). Cells were snap-frozen in liquid nitrogen and stored at −80 °C for later use.

### 4.4. Chromatin Immunoprecipitation (ChIP) and Sequencing

Cell lysates were re-suspended in ice-cold nuclear lysis immunoprecipitation (NIP) buffer containing two volumes of SDS lysis buffer and one volume of dilution buffer (100 mM NaCl, 50 mM Tris-Cl, 5 mM EDTA, 0.02% NaN^3^, 5% Triton X-100; Millipore Sigma). Cell lysates were sonicated to obtain chromatin fragments ranging from 100 to 300 base pairs (bp). The sonicator (130 watt ultrasonic sonicator, Sonics, Newtown, CT, USA) was set to 55% power, with alternating time intervals of 30 s “ON” and 30 s “OFF” for a total of 20 min “ON” time for FA crosslinked cells (one-step) or a total of 40 min “ON” time for cells dual crosslinked with DSG and FA (two-step). To verify optimum DNA size fragmentation and concentration, phenol/chloroform DNA extraction and ethanol precipitation were performed (see section below), and purified DNA was run on a 1% agarose gel (Millipore Sigma). The 50–70 μg/mL of cell lysate was transferred into 1.5 mL tubes (Eppendorf, Hamburg, Germany) and centrifuged at 14,000× *g* for 30 min. Debris-free lysate was transferred to 1.5 mL safe-lock tubes (Eppendorf), 1% of the lysate was set aside for input control and the remaining lysate was immunoprecipitated overnight at 4 °C with antibodies: BAF170 (Cell SignalingTechnology, Danvers, MA, USA, Cat: 12760S), BAF155 (Cell Signalling, Cat: 11956S), BRG1 (Abcam, Cambridge, United Kingdom, Cat: ab110641), SS18 (Cell Signalling, Cat: 21792S), SS18L (CREST; Proteintech Rosemont, IL, USA, Cat: 12439-1-AP), ARID1A (SantaCruz, Dallas, TX, USA, Cat: sc-373784), BRD9 (ActiveMotif, Carlsbad, CA, USA, Cat: 61537), BAF180 (Bethyl Laboratories, Montgomery, TX, Cat: A301-591A), ARID2 (AbioCode, Agoura Hills, CA, USA, Cat: 2380-1), BRD7(Cell Signalling, Cat: 14910S), HA-tag (Cell Signalling, Cat: 3724S), H3K27Me3 (MilliporeSigma, Burlington, MA, Cat: 07-449), IgG (Merck-Millipore, Cat: 12-340, H3K27ac (Merck-Millipore, Cat: 07-360), H3K4me1 (Abcam, Cat: ab8895). Immune-complexes were incubated with 70–100 μL pre-washed protein A or G Dynabeads (Invitrogen) on a rotator for 4 h at room temperature or overnight at 4 °C. Beads were washed with 1 mL of the following buffers for 5 min at 4 °C: Mixed Micelle Buffer (3 times), Buffer 500 (2 times) and LiCl Detergent Wash Buffer (2 times). The last wash was performed in TE buffer for 2 min at 4 °C. ChIP elution buffer (1% SDS, 1 mM NaHCO^3^) was added to the beads and incubated at 65 °C for 1 h. The beads were pelleted on a magnetic rack and the supernatant was incubated overnight at 65 °C to reverse the crosslinking. The eluate was phenol/chloroform extracted and ethanol precipitated. The DNA pellet was resuspended in 30–50 μL TE buffer (1 mM Tris-HCl pH 8, 0.1 mM EDTA) and transferred to a new Lo-Bind 1.5 mL tube, and stored at −20 °C.

### 4.5. Phenol/Chloroform-Extraction and Ethanol Precipitated

Sonicated and input DNA was RNase (1μg/mL, Thermo Fisher Scientific) and Phosphatase K (1 μL/mL, Thermo Fisher Scientific) treated for 20 min at 37 °C and for 1 h at 55 °C, respectively. DNA was diluted in TE buffer and one volume of phenol/chloroform (Invitrogen) was added. Samples were vortexed and centrifuged for 10 min at 14,000× *g*. The upper aqueous phase was transferred to a new low-bind Eppendorf tube and 1 mL ethanol containing 40 μL 3 M sodium acetate (pH 5.5) and 2 μL glycogen (Millipore Sigma) was added. DNA was precipitated at −80 °C for 30 min and pelleted by centrifugation at 14,000× g for 20 min. Contaminants were removed with 80% ethanol and the DNA pellet was dried using a vacuum concentrator (Thermo Savant DNA 110 SpeedyVac). DNA pellets were re-suspended in TE buffer.

### 4.6. Library Preparation and Sequencing

Before library preparation and sequencing, all antibodies were first screened for ChIP-grade potential in SMARCB1-expressing cells by ChIP-qPCR. We considered antibodies suitable for ChIP-seq when target enrichment at positive control genes was 10-fold greater than IgG background signal at the same loci (Figure S4, IGV screenshot of antibodies used for ChIP-seq). ChIP-DNA was quantified using the Qubit^®^ 2.0 Fluorometer (Thermo Fisher Scientific). ChIP libraries were prepared from 10 ng DNA using the NEBNext^®^Ultra-library preparation kit for Illumina (New England BioLabs, Ipswich, MA, USA) with Dual indexing NEBNext^®^ Multiplex Oligos (Dual Index Primers Set 1) following the manufacturer’s protocol. DNA libraries were purified and size selected (200–500 base pairs) by two rounds of AMPure bead purification. Library fragment length was validated using the High Sensitivity (HS) ScreenTape for 2200 TapeStation (Agilent, Santa Clara, CA, USA). ChIP library amplification was validated by qPCR at a set of known positive and negative control genes.

### 4.7. ChIP-Seq Data Analysis

Paired-end FastQ files were downloaded from the sequencing facility’s website and processed through FastQC [68] for quality control. Bowtie2 version 2.1.0 [69] was used to map the reads against the hg19 human genome assembly. The ‘--local’ parameter was applied to carry out soft-trimming. Any reads not considered the primary alignment or without mapped mate were excluded by using samtools (version 1.3.1) [70] with the ‘view’ command and flag ‘-F 268′. PCR duplicates were removed through the samtools ‘rmdup’ command. Peak detection was carried out with MACS2 version 2.2.6 [71], using the ‘callpeak’ command with ‘hs’ as the genome and both treatment and control specified. To identify differential regions we followed the steps outlined in https://github.com/macs3-project/MACS/wiki/Call-differential-binding-events (accessed 25 September 2019) This involves running the MACS2 sub commands predictd, callpeak, and bdgdiff. This results in three sets of regions, either specific to one or the other sample, or common to both. To filter out irrelevant differential binding, we then filtered the determined regions by intersecting with the summits of peaks called by MACS2. For generation of heatmaps, the deepTools package (version 3.3.0) [72] was used. With the help of the ‘bamCompare’ command, the controls were subtracted from the ChIP tracks. The resulting BigWig files were normalized using the RPKM option. Other options applied were ‘--binSize 1′, ‘--extendReads’ and ‘--centerReads’ for highest possible resolution and sharper signals. The ‘computeMatrix’ command was used together with the blacklist provided by http://mitra.stanford.edu/kundaje/akundaje/release/blacklists/hg19-human/ (accessed 9 November 2018) to exclude problematic regions. Plots of heat maps were generated through the ‘plotHeatmap’ command (also part of the deepTool package). The Homer software (version 4.10) was used to detect superenhancers and annotate peak regions. Homer “findMotifsGenome” was used to identify enriched transcription factor binding motifs from input bed files. Bedtools [73] was used to detect unique and common peaks across datasets. Statistical analysis was performed using Prism 8 software.

#### Identification of Kidney-Specific Enhancers

Enhancer regions from 10 unrelated tissues (kidney, adrenal, brain, breast, heart, liver, lung, ovary, placenta, skeletal muscle) were downloaded together with tissue annotation from the tissue-specific enhancer database, TiED [26]. TiED kidney-specific enhancers (*n* = 2915) and brain-specific enhancers (*n* = 3637) were identified as overlapping H3K27ac and H3K4me1 peaks unique to kidney tissue and absent from the other 9 tissue types.

### 4.8. DNA Methylation Analysis

The signal intensities for dataset GSE44847, which was generated with the Illumina HumanMethylation27 BeadChip, were downloaded from Gene Expression Omnibus. For each probe an M-value was calculated by transferring the ratio of methylated vs. unmethylated signal into log2 space. We used three replicates each of samples labelled as ‘rhabdoid tumors of the kidney’ (RTK) and ‘non-neoplastic kidney’ (NK), and merged them via the mean average. The merged M-values of RTK and NK were plotted against each other using ggplot2 functions in R. Probes with high M-value in RTK samples and low M-value in NK samples were filtered and annotated. To do this, the bead locations were extracted from GEO accession GPL8490 and transferred from hg18 to hg19 using UCSC’s LiftOver tool (https://genome.ucsc.edu/cgi-bin/hgLiftOver, accessed on 15 March 2021). The tool annotatePeaks.pl from the Homer package was applied to assign the nearest gene to each probe.

To correlate methylation data with gene expression, RNA-seq read counts for rhabdoid tumor and matched normal kidney samples were downloaded from the NCBI’s TARGET website [34]. Only genes that had at least 0.5 counts per million in one or more libraries were kept. A pseudocount of 4 was added to the remaining entries before they were normalized with DESeq2. Probes from the methylation array were annotated with Homer to derive the closest gene. These genes were cross-referenced with the RNA-seq results to select the cohort of genes associated with a set of methylated probes in MRTK tumors. Boxplots of normalized and log2-transformed counts were generated with R.

### 4.9. RNA-Seq Data Analysis

RNA-seq data for G401 were downloaded in the form of read counts from GEO series GSE90633. We used two replicates each of samples labelled ‘G401_Empty_Day3′ and ‘G401_BAF47_Day3′. For BT16, three replicates each of samples labelled ‘BT16_NoDox’ and ‘BT16_Dox’ were downloaded from GEO series GSE71505 [23,24]. Differential expression analysis was carried out with DESeq2 [74].

## 5. Conclusions

We propose that the observed apparent redundancy in epigenetic silencing through both histone/chromatin modification as well as promoter DNA methylation serves to provide credence to our theory of MRTK representing a classic example wherein an aggressive primitive cancer results from dysregulated proliferation coupled with arrested differentiation.

## Data Availability

The data presented in this study are openly available in GEO (https://www.ncbi.nlm.nih.gov/geo/, 14 May 2021), GEO accession number GSE174446.

## Supplementary Materials

The following are available online at https://www.mdpi.com/article/10.3390/cancers13112561/s1, Figure S1: Extended Western blot showing SMARCB1 expression in BT16 HA-SMARCB1 cells. Figure S2: Extended Western blot showing SMARCB1 expression in G401 HA-SMARCB1 cells: Figure S3: Super-Enhancers scatter plot, based on log2-scaled average RPKM normalised, input-subtracted signal strength across each super-enhancer region in mock- and doxycycline treated BT16 and G401 cell lines, Figure S4: IGV screenshot of antibodies used to target SWI/SNF, PBAF and ncBAF by ChIP-seq, Table S1: Homer motif analysis of BRD9 targets in BT16 cells, Table S2: Homer motif analysis of BRD9 targets in G401 cells, Table S3: Promoter methylation m-values in normal kidney, Table S4: Promoter methylation m-values in Rhabdoid tumors.
