# Supplementary material for: Global Chromatin Changes Resulting from Single-Gene Inactivation—The Role of SMARCB1 in Malignant Rhabdoid Tumor
Source: Cancers (Basel). 2021 May 23;13(11):2561. doi: 10.3390/cancers13112561 (PMC8197137; doi:10.3390/cancers13112561)
Supplement: Supplementary file 1 [file cancers-13-02561-s001.zip › Supplemental FigS1_S4.pptx]

## Slide 1
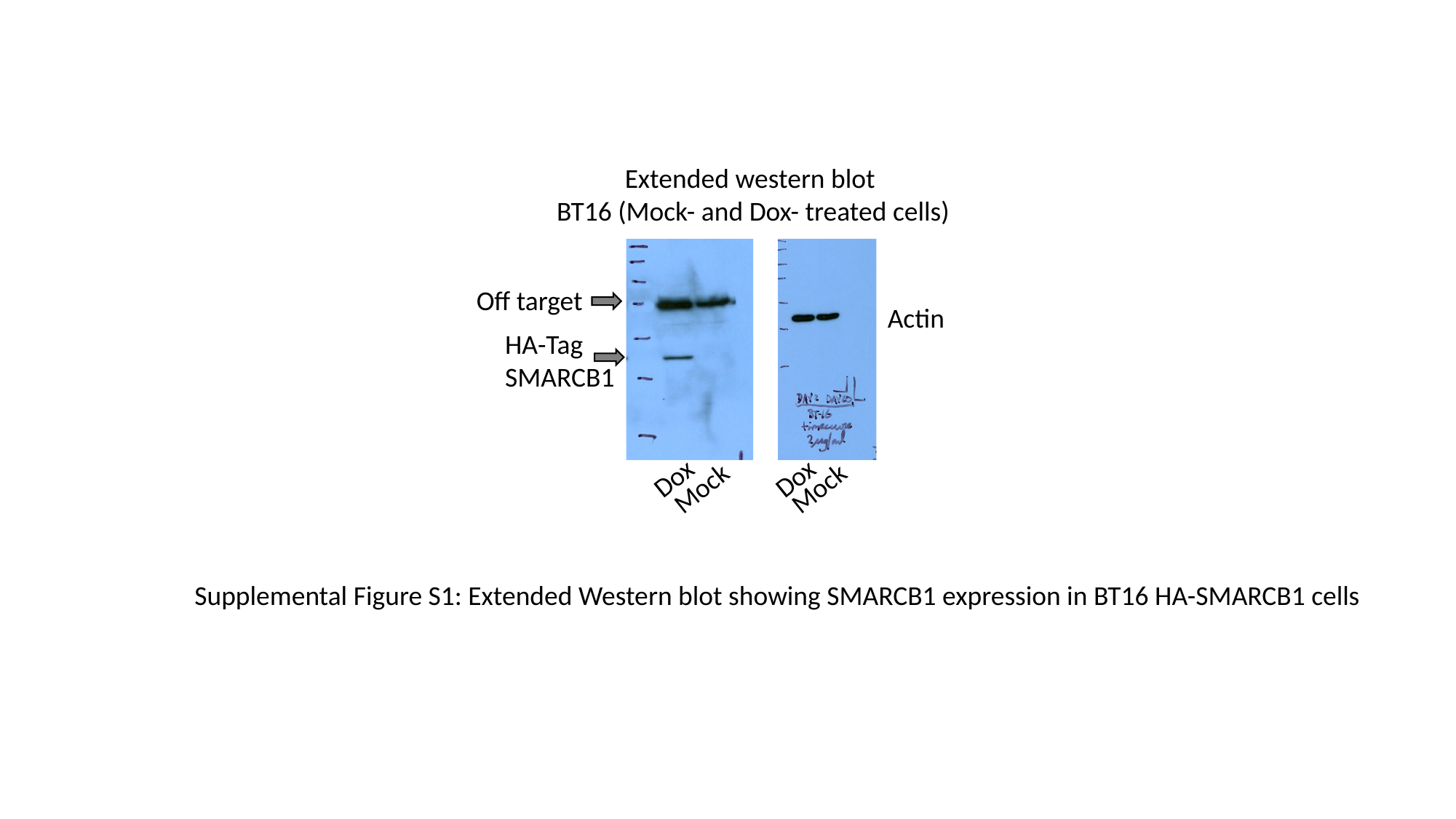

Extended western blot
BT16 (Mock- and Dox- treated cells)
Off target
Actin
HA-Tag
SMARCB1
Dox
Dox
Mock
Mock
Supplemental Figure S1: Extended Western blot showing SMARCB1 expression in BT16 HA-SMARCB1 cells

## Slide 2
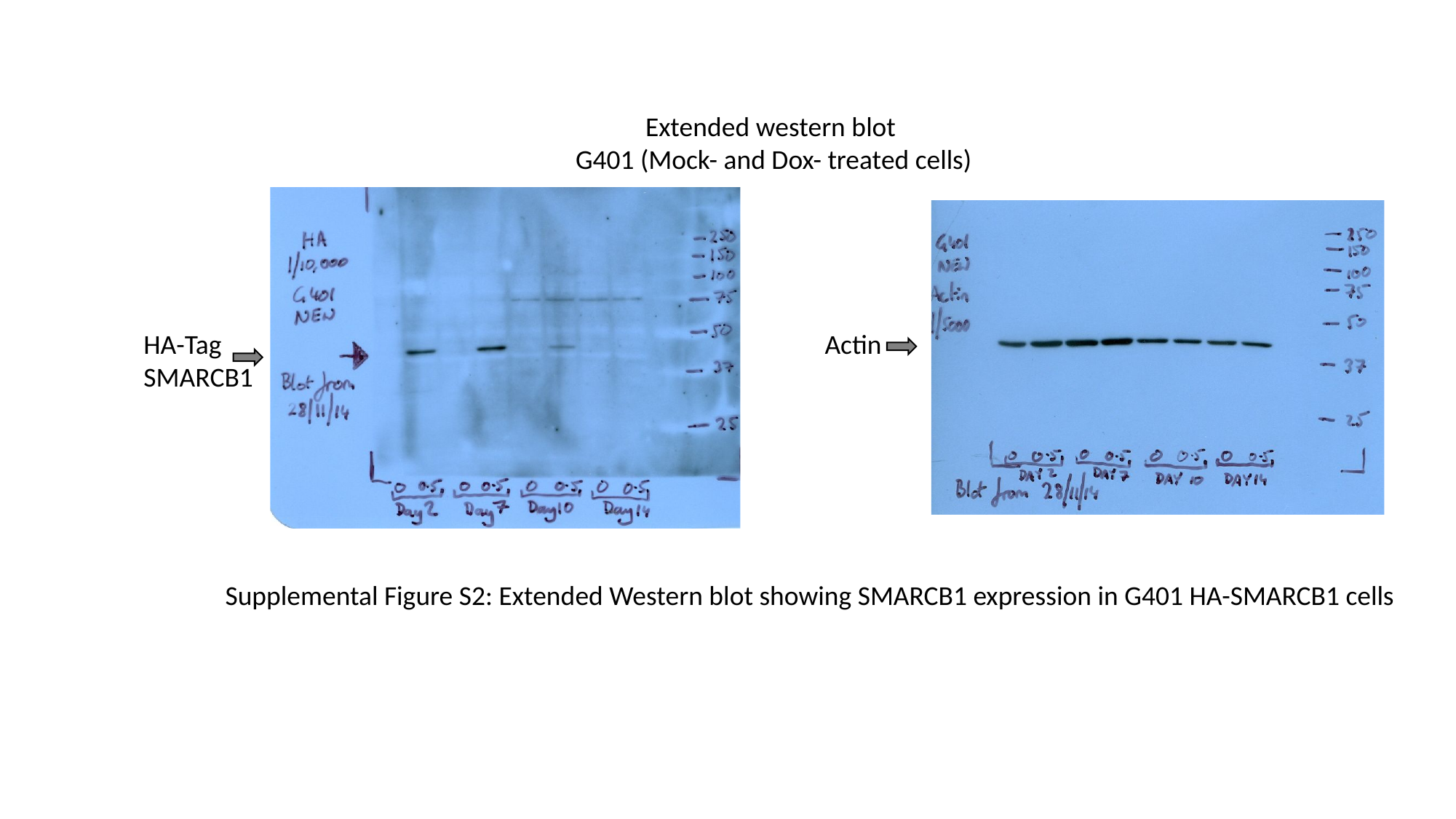

Extended western blot
G401 (Mock- and Dox- treated cells)
HA-Tag
SMARCB1
Actin
Supplemental Figure S2: Extended Western blot showing SMARCB1 expression in G401 HA-SMARCB1 cells

## Slide 3
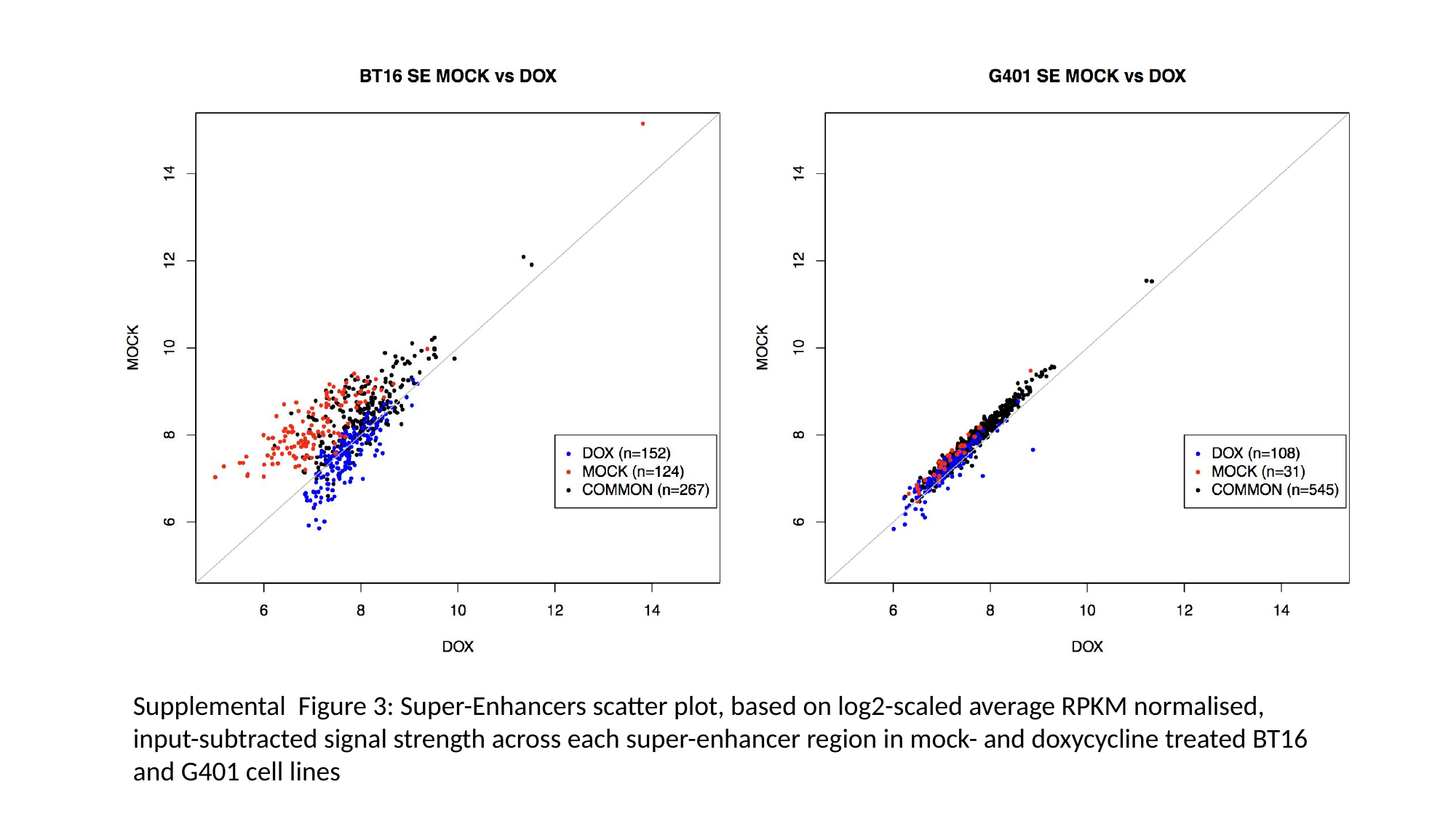

Supplemental Figure 3: Super-Enhancers scatter plot, based on log2-scaled average RPKM normalised, input-subtracted signal strength across each super-enhancer region in mock- and doxycycline treated BT16 and G401 cell lines

## Slide 4
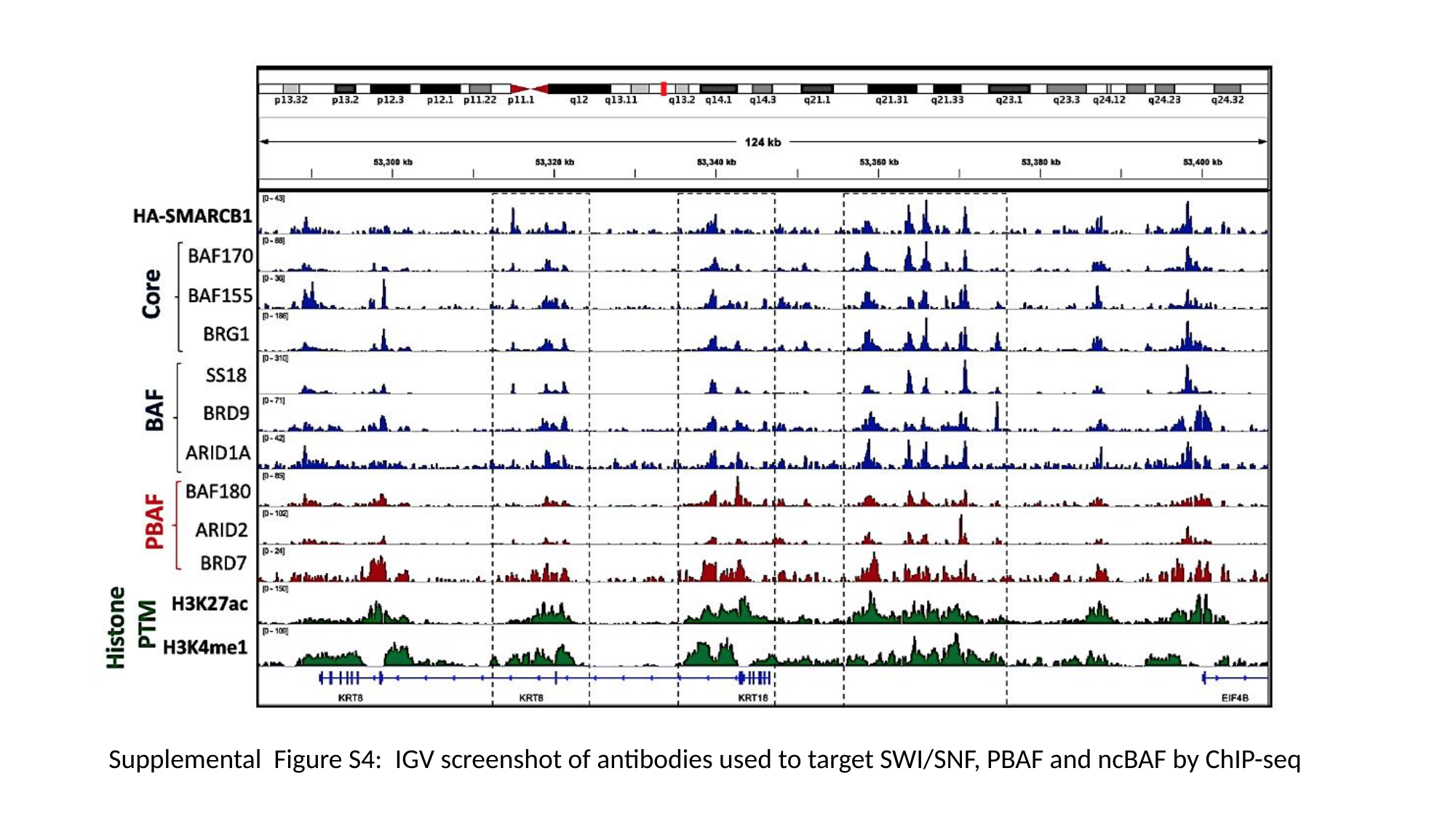

Supplemental Figure S4: IGV screenshot of antibodies used to target SWI/SNF, PBAF and ncBAF by ChIP-seq
